# OpenSAFELY NHS Service Restoration Observatory 2: changes in primary care clinical activity in England during the COVID-19 pandemic

**DOI:** 10.3399/BJGP.2022.0301

**Published:** 2023-04-18

**Authors:** Helen J Curtis, Brian MacKenna, Milan Wiedemann, Louis Fisher, Richard Croker, Caroline E Morton, Peter Inglesby, Alex J Walker, Jessica Morley, Amir Mehrkar, Sebastian CJ Bacon, George Hickman, David Evans, Tom Ward, Simon Davy, William J Hulme, Orla Macdonald, Robin Conibere, Tom Lewis, Martin Myers, Shamila Wanninayake, Kiren Collison, Charles Drury, Miriam Samuel, Harpreet Sood, Andrea Cipriani, Seena Fazel, Manuj Sharma, Wasim Baqir, Chris Bates, John Parry, Ben Goldacre, The OpenSAFELY Collaborative

**Affiliations:** The Bennett Institute for Applied Data Science, Nuffield Department of Primary Care Health Sciences, University of Oxford, Oxford.; The Bennett Institute for Applied Data Science, Nuffield Department of Primary Care Health Sciences, University of Oxford, Oxford.; The Bennett Institute for Applied Data Science, Nuffield Department of Primary Care Health Sciences, University of Oxford, Oxford.; The Bennett Institute for Applied Data Science, Nuffield Department of Primary Care Health Sciences, University of Oxford, Oxford.; The Bennett Institute for Applied Data Science, Nuffield Department of Primary Care Health Sciences, University of Oxford, Oxford.; The Bennett Institute for Applied Data Science, Nuffield Department of Primary Care Health Sciences, University of Oxford, Oxford.; The Bennett Institute for Applied Data Science, Nuffield Department of Primary Care Health Sciences, University of Oxford, Oxford.; The Bennett Institute for Applied Data Science, Nuffield Department of Primary Care Health Sciences, University of Oxford, Oxford.; The Bennett Institute for Applied Data Science, Nuffield Department of Primary Care Health Sciences, University of Oxford, Oxford.; The Bennett Institute for Applied Data Science, Nuffield Department of Primary Care Health Sciences, University of Oxford, Oxford.; The Bennett Institute for Applied Data Science, Nuffield Department of Primary Care Health Sciences, University of Oxford, Oxford.; The Bennett Institute for Applied Data Science, Nuffield Department of Primary Care Health Sciences, University of Oxford, Oxford.; The Bennett Institute for Applied Data Science, Nuffield Department of Primary Care Health Sciences, University of Oxford, Oxford.; The Bennett Institute for Applied Data Science, Nuffield Department of Primary Care Health Sciences, University of Oxford, Oxford.; The Bennett Institute for Applied Data Science, Nuffield Department of Primary Care Health Sciences, University of Oxford, Oxford.; The Bennett Institute for Applied Data Science, Nuffield Department of Primary Care Health Sciences, University of Oxford, Oxford.; The Bennett Institute for Applied Data Science, Nuffield Department of Primary Care Health Sciences, University of Oxford, Oxford.; Beacon Medical Group, Plymouth.; Royal Devon University Healthcare NHS Foundation Trust, Barnstaple.; Lancashire Teaching Hospitals NHS Foundation Trust, Preston.; The Manor Surgery, Oxford.; NHS England and NHS Improvement, London.; Herefordshire and Worcestershire Health and Care NHS Trust, Worcester.; Wolfson Institute of Population Health, Queen Mary University of London, London.; University College London Hospitals NHS Foundation Trust, London.; Department of Psychiatry, University of Oxford, Oxford.; Department of Psychiatry, University of Oxford, Oxford.; Department of Primary Care and Population Health, University College London, London.; NHS England and NHS Improvement, London.; TPP, Leeds.; TPP, Leeds.; The Bennett Institute for Applied Data Science, Nuffield Department of Primary Care Health Sciences, University of Oxford, Oxford.

**Keywords:** COVID-19, electronic health records, general practice, primary health care

## Abstract

**Background:**

The COVID-19 pandemic has disrupted healthcare activity across a broad range of clinical services. The NHS stopped non-urgent work in March 2020, later recommending services be restored to near-normal levels before winter where possible.

**Aim:**

To describe changes in the volume and variation of coded clinical activity in general practice across six clinical areas: cardiovascular disease, diabetes, mental health, female and reproductive health, screening and related procedures, and processes related to medication.

**Design and setting:**

With the approval of NHS England, a cohort study was conducted of 23.8 million patient records in general practice, in situ using OpenSAFELY.

**Method:**

Common primary care activities were analysed using Clinical Terms Version 3 codes and keyword searches from January 2019 to December 2020, presenting median and deciles of code usage across practices per month.

**Results:**

Substantial and widespread changes in clinical activity in primary care were identified since the onset of the COVID-19 pandemic, with generally good recovery by December 2020. A few exceptions showed poor recovery and warrant further investigation, such as mental health (for example, for ‘Depression interim review’ the median occurrences across practices in December 2020 was down by 41.6% compared with December 2019).

**Conclusion:**

Granular NHS general practice data at population-scale can be used to monitor disruptions to healthcare services and guide the development of mitigation strategies. The authors are now developing real-time monitoring dashboards for the key measures identified in this study, as well as further studies using primary care data to monitor and mitigate the indirect health impacts of COVID-19 on the NHS.

## INTRODUCTION

The COVID-19 pandemic disrupted healthcare services globally.^1^ In March 2020, NHS England initially promoted measures to reduce viral transmission and provide only essential health services.^2^^,^^3^ The World Health Organization (WHO) recommended rapid assessments of healthcare capacity and the development of key performance indicators.^4^^,^^5^ From August 2020, NHS England aimed to restore primary care and other services back to normal activity where clinically appropriate.^6^

Various studies have assessed the impact of the pandemic on non-COVID health services. For example, a review of the impact on mental health found associations with adverse psychiatric symptoms and a significant difference was found in women reporting difficulties accessing contraception; significant differences in primary care prescribing patterns have been observed.^7^^–^^10^ Disruption was expected more widely, in areas such as health screening, where national screening programmes for bowel and breast cancer paused invitations in March 2020.^11^ In a survey of healthcare professionals from 47 countries, diabetes, chronic obstructive pulmonary disease (COPD), and hypertension were found to be the most impacted conditions as a result of disruption to routine care.^12^ These conditions are also associated with higher risk of morbidity and mortality from COVID-19.^13^^–^^16^ It is likely that reduction in routine care for chronic diseases, such as cardiovascular disease (CVD), which are the largest cause of morbidity globally, will contribute to a major burden of indirect effects of the pandemic.^17^ This emphasises the importance of maintaining good routine care as part of the post-COVID-19 recovery.

**Table:** How this fits in

During the COVID-19 pandemic, routine healthcare services in England faced significant disruption, and NHS England recommended restoring NHS services to near-normal levels before winter 2020. The authors’ previous report covered the disruption and recovery in pathology tests and respiratory activity. In this report an additional six areas of common primary care activity are described. The study found that most activities exhibited significant reductions during the first wave of the pandemic (with most recovering to near-normal levels by December 2020); however, many important aspects of care — especially those of a more time-critical nature — were maintained throughout the pandemic. As such, the authors recommend key measures for ongoing monitoring and further investigation to help measure and mitigate the ongoing indirect health impacts of COVID-19 on the NHS.

The authors previously reported a data-driven approach for monitoring healthcare disruptions and recovery using the OpenSAFELY platform (https://opensafely.org) combined with input from a clinical advisory group, which intended to explore changes in high-volume areas, including those that might otherwise be missed.^18^ Pathology tests and respiratory conditions were selected as key examples and results showed that activity decreased substantially during the first national lockdown in March 2020 but subsequently recovered to near-normal levels by July 2020. However, it was also found that some activities such as blood coagulation tests were well-maintained, suggesting that important clinical care was effectively prioritised.^18^

This study aims to generate an overall picture of changes in clinical activity in primary care across key areas of medicine; and to identify key measures to continuously monitor the impacts of COVID-19 on the NHS and inform further studies. Building on the previous study,^18^ the time frame was extended to December 2020 and the activities analysed were expanded to cover six further clinical areas: CVD, diabetes, mental health, female and reproductive health, screening and related procedures, and processes related to medication. These areas were selected as they were likely to include a high volume of activity, representing a large burden of diseases, that is relevant and actionable to front-line practice as well as being potentially indicative of wider problems in service delivery across the NHS during the COVID-19 pandemic.

## METHOD

### Study design and data source

Following the methodology previously described,^18^ a cohort study was conducted using routinely collected pseudonymised primary care electronic health records (EHR) in the OpenSAFELY-TPP platform (https://opensafely.org). This included 40% of England’s general practices (*n* = 2500, those using TPP SystmOne software), covering 24 million patients. In brief, these structured general practice data include one record for every diagnostic code, prescription, blood test, investigation, or similar in primary care.

### Study population and data processing

The study population and data processing were as described in the authors’ previous study,^18^ except for a later study end date (31 December 2020) and inclusion of all patients registered with a general practice on 31 December 2020. Occurrences of all coded clinical activities per month throughout 2019 to 2020 were counted, by Clinical Terms Version 3 (CTV3) code (including diagnoses, investigations, and other clinical/administrative processes but excluding medications/vaccinations), grouped by general practice, with the exception of Structured Medication Reviews (SMRs), which were counted using a newly introduced SNOMED-CT code.^19^ CTV3 is a coding dictionary with a tree structure with ‘Parent’ concepts describing broad clinical areas and ‘Child’ codes of increasing specificity as you descend the hierarchy, which enabled grouping of related codes into clinical topics. More information on the use of CTV3 codes can be found in the authors’ previous report.^18^ Any codes with <1000 total occurrences in 2020 were excluded in order to identify the most frequent clinical activities. The CTV3 parent–child hierarchy was used to group similar activities, and each code was mapped to high-level CTV3 concepts to assist with categorisation into broad topics (for example, ‘cardiovascular’). The monthly rate of code usage per 1000 registered patients and the median and deciles across practices were calculated for each code/group.

### Study measures

Activity and selected clinical codes were grouped by relevance to each of the following topics: CVD, diabetes, mental health, female and reproductive health, screening and related procedures, and processes related to medication. Prescribing, which is coded in GP systems using the NHS dictionary of medicines and devices, was not included in this study as high-quality, routinely updated analysis of primary care dispensing data are already openly available through OpenPrescribing (https://openprescribing.net). The selection of clinical codes was largely based on existing CTV3 concepts and keyword searching. A detailed description of the methodology is available in Supplementary Information S1 and Supplementary Table S1.

### Clinical advisory group

A clinical advisory group was established to review the findings. The group was formed by invitation of clinicians known through existing professional relationships and consisted of GPs, pharmacists, pathologists, other relevant specialists, and national clinical advisors. The raw results of the data-driven approach on each topic were discussed with the advisory group during a series of online meetings to prioritise clinical topics and inform interpretation. The group also had the opportunity to comment on these documents outside of the meetings and arrange further meetings with the research team if needed. Additionally, the group was asked to select ‘key measures’ of activity from each clinical area that would benefit from routine monitoring and targeted action.

### Software and reproducibility

Data management and analysis were performed using Python 3.8. Code for data management and analysis is available online (https://github.com/opensafely/restoration-observatory-data-driven).

### Patient and public engagement

This analysis relies on the use of large volumes of patient data. Ensuring patient, professional, and public trust is therefore of critical importance. Maintaining trust requires being transparent about the way OpenSAFELY works and ensuring that patient and public voices are represented in the design and use of the platform. Between February and July 2022, a 6-month pilot of patient and public involvement and engagement was conducted, designed to be aligned with the principles set out in the Consensus Statement on Public Involvement and Engagement with Data-Intensive Health Research.^20^ Engagement was focused on the broader OpenSAFELY platform and comprised three sets of activities: explain and engage, involve and iterate, and participate and promote.

- To explain and engage, a public website was developed (https://opensafely.org) that provides a detailed description of the OpenSAFELY platform in language suitable for a lay audience. An accompanying explainer video is also being developed.
- To involve and iterate, the OpenSAFELY ‘Digital Critical Friends’ Group was created; comprised of approximately 12 members representative in terms of ethnicity, gender, and educational background. This group has met every 2 weeks to engage with and review the OpenSAFELY website, governance process, principles for researchers, and FAQs.
- To participate and promote, the authors are conducting a systematic review of the key enablers of public trust in data-intensive research and have participated in the stakeholder group overseeing NHS England’s ‘data stewardship public dialogue’.

## RESULTS

All included clinical codes/groups for each topic along with their total 2020 usage are shown in Supplementary Tables S2a‒S2f and S3. Results are summarised by topic, highlighting selected code usage that has been identified as interesting by the clinical advisory group at key points in the pandemic in England (February, April, and December 2020; Table 1).

**Table 1.** Rate of recording of selected CTV3 codes and groups of codes for each topic in February, April, and December 2020. Clinical topics include: a) cardiovascular disease; b) diabetes; c) mental health; d) female and reproductive health; e) screening and related procedures; and f) processes related to medication. Table shows median rate across general practices in England per 1000 registered patients and % change on the same month in previous year. Further detailed breakdowns of code recording are shown in Supplementary Tables S2a–S2f

**Topic**	**CTV3 code/group**	**Median rate per 1000 registered patients (% change on previous year)**		

		**February**	**April**	**December**
a) CVD	24 — ‘Examination of cardiovascular system’	164.2	19.8 (−86.9%)	82.6 (−42.6%)
	18 — ‘Cardiovascular symptoms (and [heart])’	0.6	0.3 (−57.1%)	0.4 (−17.7%)
	G5 — ‘Other forms of heart disease’	0.5	0.3 (−48.9%)	0.4 (−6.4%)
	XaQVY — ‘QRISK2 cardiovascular disease 10 year risk score’	6.9	0.6 (−98.3%)	3.0 (−38.9%)
	XaKFx — ‘Average home systolic blood pressure’	0.4	0.2 (−8.1%)	0.7 (+102.8%)
	XaKFw — ‘Average home diastolic blood pressure’	0.4	0.2 (−8.3%)	0.6 (+98.0%)

b) Diabetes	66A — ‘Diabetic monitoring’	4.6	0.4 (−87.5%)	3.3 (−16.6%)
	XaPbt — ‘Haemoglobin A1c level — IFCC standardised’	28.2	3.4 (−86.2%)	22.4 (−0.6%)
	XaIIj — ‘Diabetic retinopathy screening’	1.8	0.0 (−100.0%)	0.7 (−53.7%)
	XaIeH — ‘O/E — Right diabetic foot at low risk’	3.8	0.0 (−100.0%)	2.7 (−18.0%)
	XaIuE — ‘Diabetic foot examination’	0.2	0.0 (−100.0%)	0.2 (+113.8%)

c) Mental health	E2 — ‘Neurotic, personality, and other nonpsychotic disorders’	1.2	0.6 (−44.5%)	0.8 (−23.7%)
	E20 — ‘Neurotic disorder’	0.8	0.5 (−39.1%)	0.5 (−21.9%)
	XaK6f — ‘Depression interim review’	0.9	0.4 (−56.2%)	0.6 (−41.6%)
	XE0re — ‘Depressed mood’	0.6	0.2 (−64.7%)	0.4 (−19.9%)

d) Female and reproductive health	61 — ‘Contraception’	3.2	1.2 (−60.6%)	3.0 (+12.1%)
	15 — ‘[Gynaecological] or [obstetric] history’	1.7	0.8 (−51.3%)	1.3 (−17.2%)
	64 — ‘(Child health care)(inf feed meth) (breast/oth feed diff age)’	1.1	0.8 (−33.0%)	1.0 (−11.8%)
	K3 — ‘Disorder of breast’	0.2	0.1 (−57.9%)	0.2 (+17.8%)
	621 — ‘Patient currently pregnant’	0.9	0.7 (−26.6%)	0.8 (−8.0%)

e) Screening and related procedures	XaPVj — ‘Bowel cancer screening programme: faecal occult blood result’	6.4	3.0 (−52.1%)	6.5 (+12.0%)
	Xa8Pl — ‘Cervical smear’	4.8	0.2 (−95.6%)	3.9 (+20.9%)
	XaRBQ — ‘NHS Health Check completed’	1.8	0.0 (−100.0%)	0.0 (−100.0%)

f) Processes related to medication	XaF8d — ‘Medication review done’	22.7	14.6 (−31.0%)	18.7 (−8.2%)
	XaJKW — ‘Patient understands why taking all medication’	0.1	0.0 (−100.0%)	0.4 (+422.2%)

*CVD = cardiovascular disease. IFCC = International Federation of Clinical Chemistry and Laboratory Medicine. O/E = on examination.*

### Cardiovascular disease

**Figure 1.**
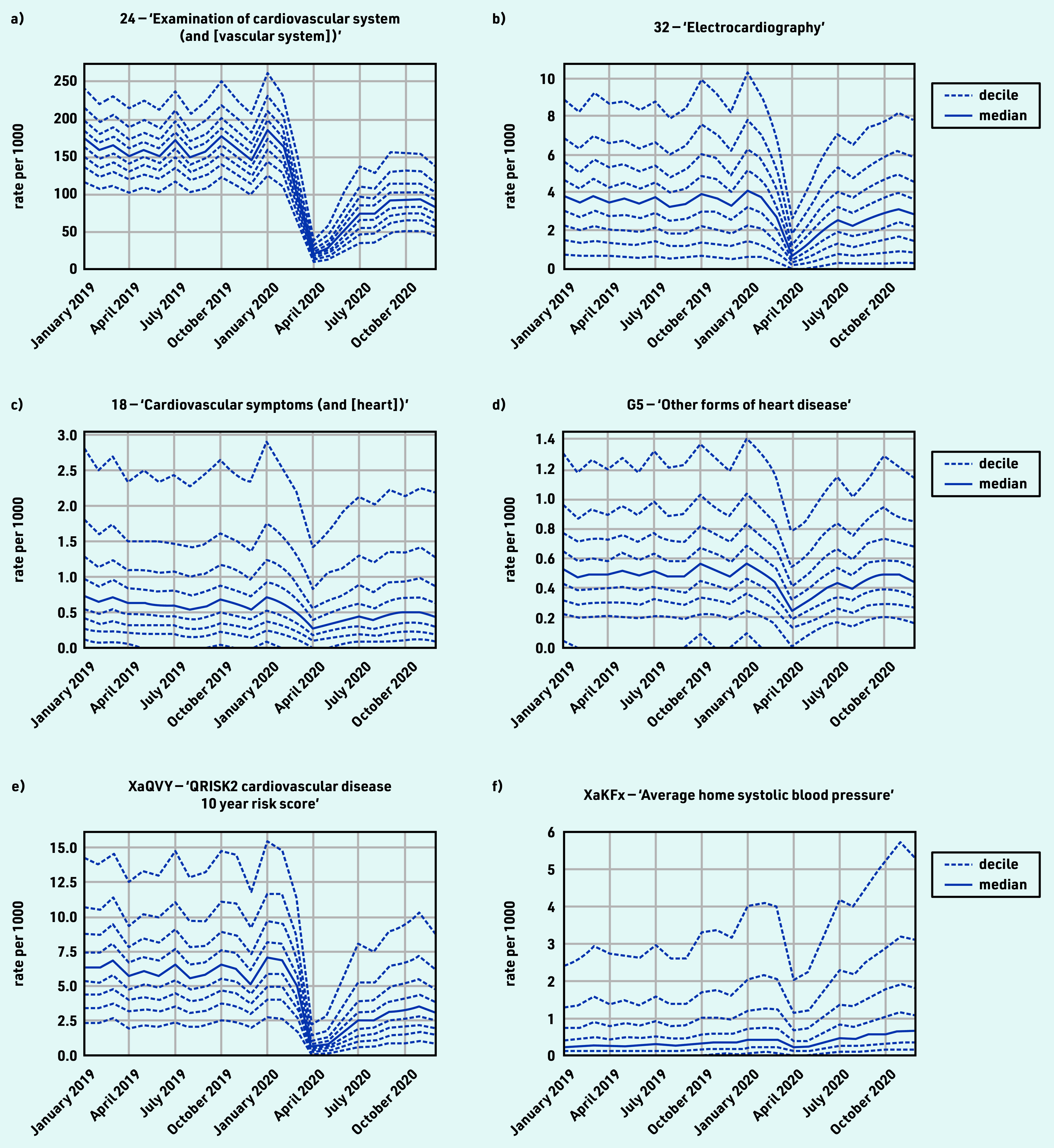
*Recording of grouped subsets of cardiovascular CTV3 codes across TPP practices in England (January 2019 to December 2020). a) ‘Examination of cardiovascular system (and [vascular system])’. b) ‘Electrocardiography’. c) ‘Cardiovascular symptoms (and [heart])’. d) ‘Other forms of heart disease’. e) ‘QRISK2 cardiovascular disease 10 year risk score’. f) ‘Average home systolic blood pressure’. Each group includes CTV3 codes that begin with the digits shown and is not necessarily an exhaustive collection of every activity related to the description.*

The majority of CVD-coded activity experienced a substantial decline during the initial stages of the pandemic, with limited recovery by September 2020 that levelled off through to December 2020, for example, ‘examination of cardiovascular system (and [vascular system])’ (including blood pressure recording) and ‘electrocardiography’ (Figures 1a and 1b). Exceptions included symptoms related to cardiovascular system (Figure 1c), which experienced a sustained drop (April −57.1% and December −17.7%); ‘other forms of heart disease’ (Figure 1d), including atrial fibrillation and heart failure, experienced a small drop but largely recovered (April −48.9% and December −6.4%); and ‘QRISK2 cardiovascular disease 10 year risk score’ (XaQVY), a CVD risk tool, dropped dramatically (April −98.3%) with limited recovery (December −38.9%). Blood pressure at home codes increased overall (December +100%; Figure 1f), but were infrequently used (total events, *n* = 410 000 systolic and *n* = 400 000 diastolic; Supplementary Table S2a) compared with blood pressure codes recorded with no setting (*n* = 11 000 000; Supplementary Table S3). For grouped codes, the top five codes represented within each group are listed in Supplementary Table S3, parent code rows a–d.

### Diabetes

**Figure 2.**
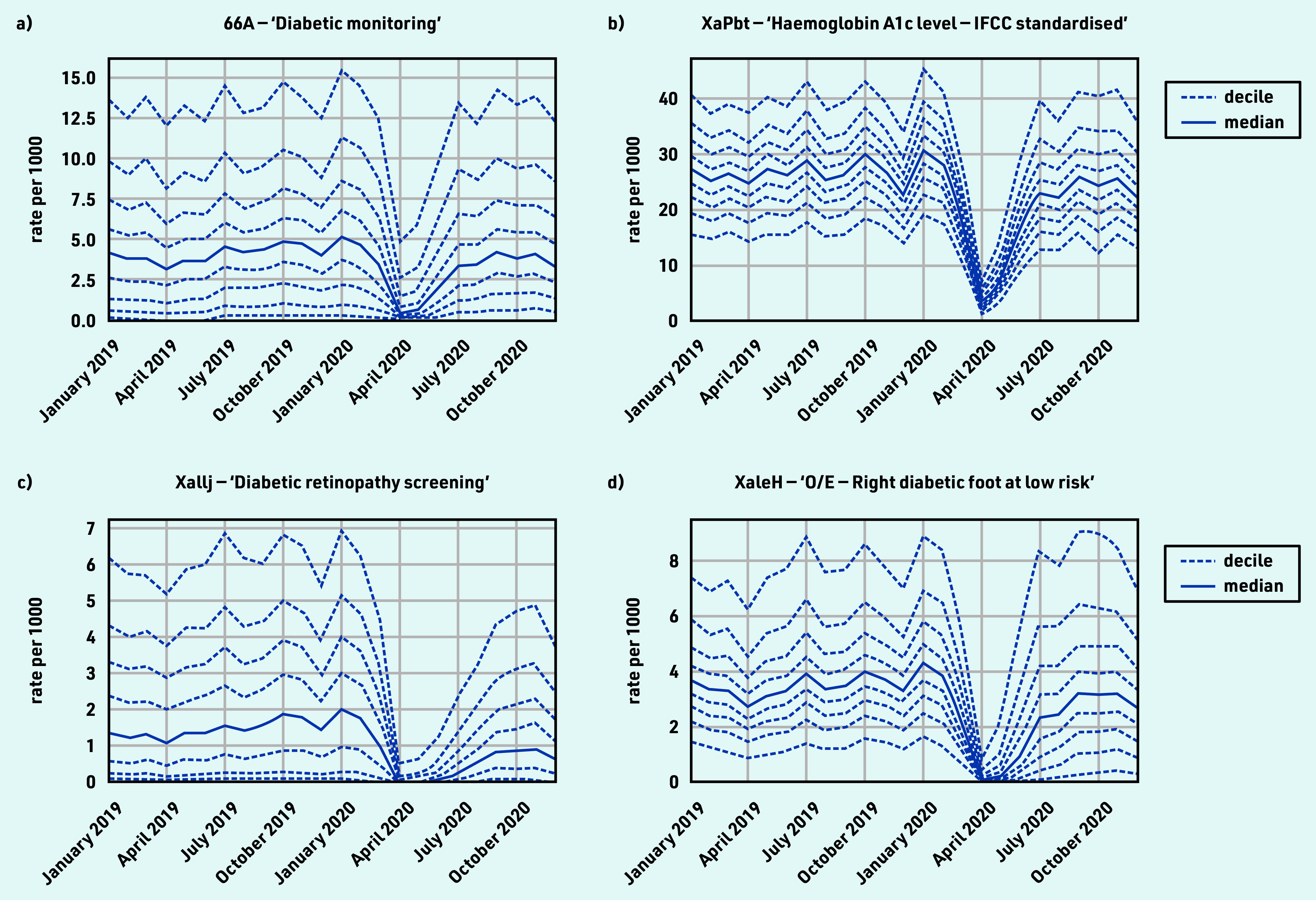
*Recording of CTV3 codes across TPP practices in England (January 2019 to December 2020). a) ‘Diabetic monitoring’. b) ‘Haemoglobin A1c level — IFCC standardised’. c) ‘Diabetic retinopathy screening’. d) ‘O/E — Right diabetic foot at low risk’. Each code is not necessarily an exhaustive collection of every activity related to the description. ‘O/E — Left diabetic foot at low risk’ is not shown but has an almost identical pattern to the right foot equivalent. Only ‘at low risk’ codes were included as codes related to moderate or increased risk did not meet the frequency threshold for the data-driven analysis.* *IFCC = International Federation of Clinical Chemistry and Laboratory Medicine. O/E = ‘on examination’.*

‘Diabetic monitoring’ and HbA1c testing both experienced a large drop with good recovery (April −87.5% and December ‒16.6%; April ‒86.2% and December ‒0.6%; Table 1b and Figures 2a and 2b). For ‘diabetic retinopathy screening’, the median dropped to zero in April, with limited recovery by December (−53.7%; Figure 2c). For ‘O/E — right diabetic foot at low risk’, the median dropped to zero in April, with good recovery by December (‒18.0%; Figure 2d). There was substantial variation in the rate of diabetes monitoring and retinopathy screening at baseline, indicating some incompleteness. For grouped codes, the top five codes represented within each group are listed in Supplementary Table S3, parent code row e.

### Mental health

**Figure 3.**
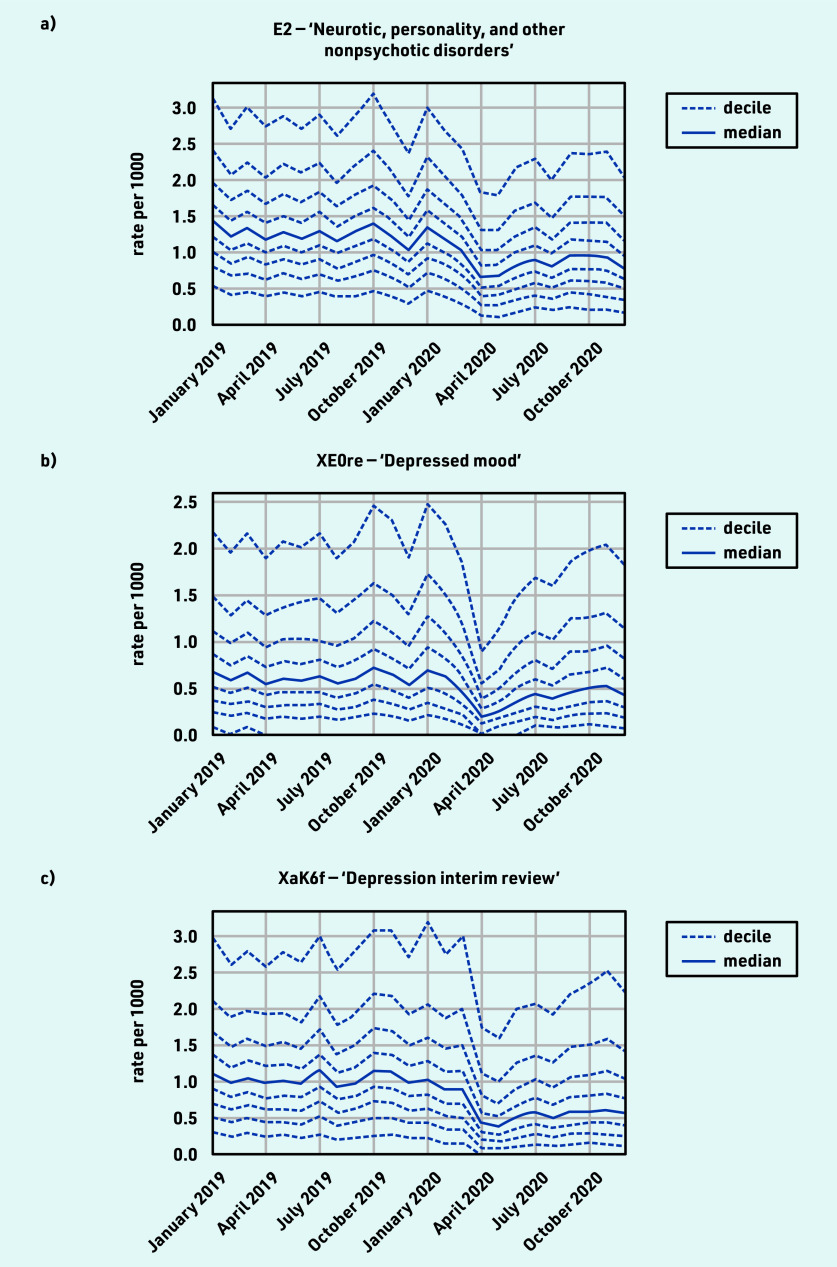
*Recording of CTV3 codes across TPP practices in England (January 2019 to December 2020). a) ‘Neurotic, personality, and other nonpsychotic disorders’. b) ‘Depressed mood’. c) ‘Depression interim review’. Each code is not necessarily an exhaustive collection of every activity related to the description.*

The majority of mental health coded activity experienced a moderate decline during the initial stages of the pandemic, with incomplete recovery by December 2020 (Table 1c), for example, ‘Neurotic, personality, and other nonpsychotic disorders’ (April −44.5% and December −23.7%; Figure 3a); ‘Depressed mood’ (April −64.7% and December −19.9%; Figure 3b). ‘Depression interim review’ activity showed particularly poor recovery (April −56.2% and December −41.6%; Figure 3c). For grouped codes, the top five codes represented within each group are listed in Supplementary Table S3, parent code row f.

### Female and reproductive health

**Figure 4.**
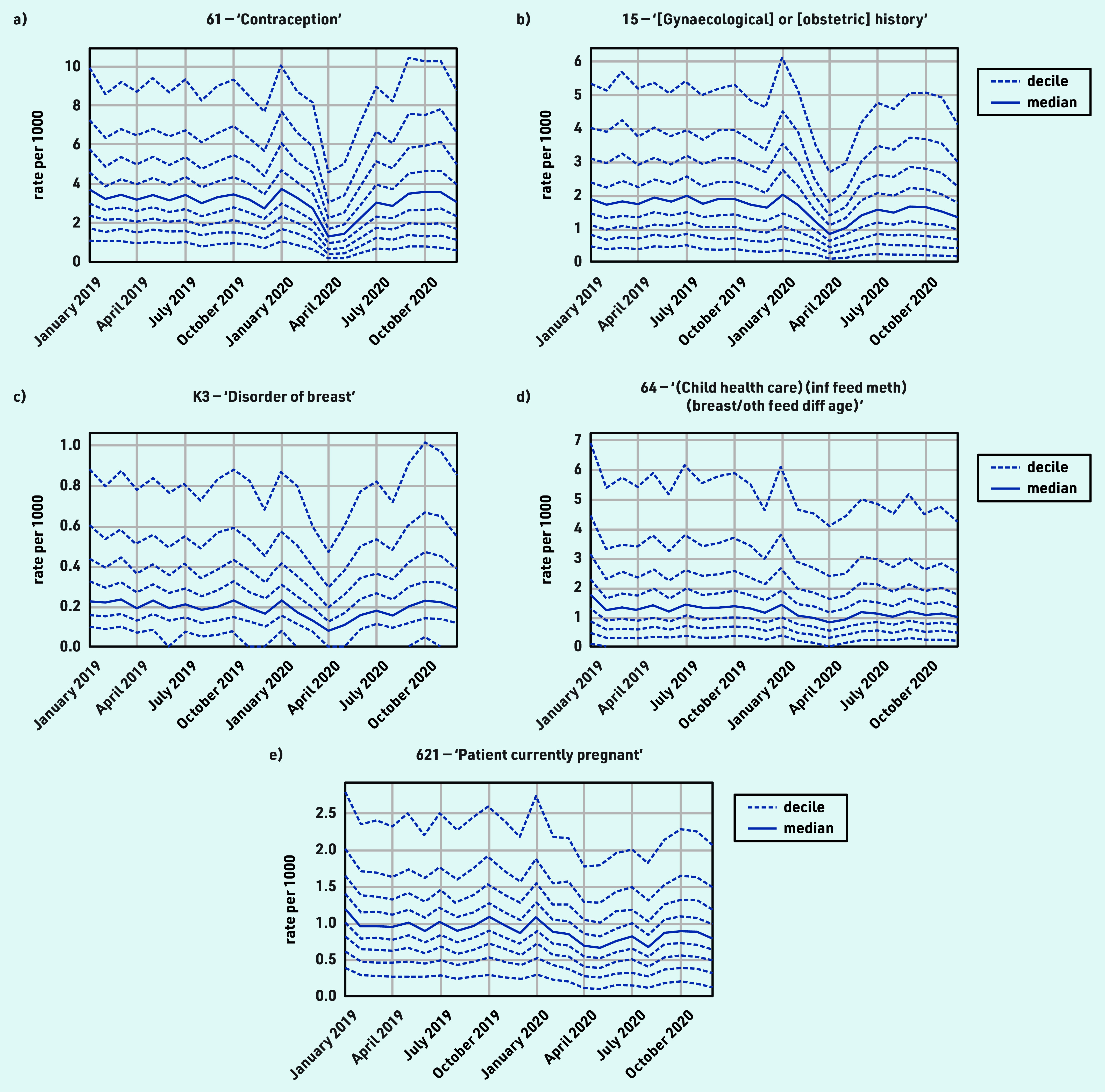
*Recording of grouped subsets of CTV3 codes related to female and reproductive health across TPP practices in England (January 2019 to December 2020). a) ‘Contraception’. b) ‘[Gynaecological] or [obstetric] history’. c) ‘Disorder of breast’. d) ‘Information about feeding methods (breast/other) at different ages — (child health care) (inf feed meth) (breast/oth feed diff age)’. e) ‘Patient currently pregnant’. Each CTV3 code does not necessarily represent all activity related to the description. breast/oth feed diff age = breast or other feeding method at different ages. inf feed meth = infant feeding method.*

The majority of female and reproductive health activity experienced a moderate decline during the initial stages of the pandemic, with either recovery to near-normal levels by December 2020, or a return to an existing increasing or decreasing trend, for example, ‘Contraception’ (codes beginning ‘61’): April −60.6% and December +12.1% (Figure 4a and Table 1d).

‘[Gynaecological] or [obstetric] history’ showed a broadly similar pattern but with a slight sustained reduction (December −17.2%; Figure 4b). Various codes encompassed gynaecological and obstetric procedures and symptoms, which generally recovered to pre-pandemic levels, although many had a median of zero throughout the period. One example was ‘Disorder of breast’ (Figure 4c, the most common code within which was ‘Breast lump’), which had a small increase by December (April −57.9% and December +17.8%).

### Screening and related procedures

**Figure 5.**
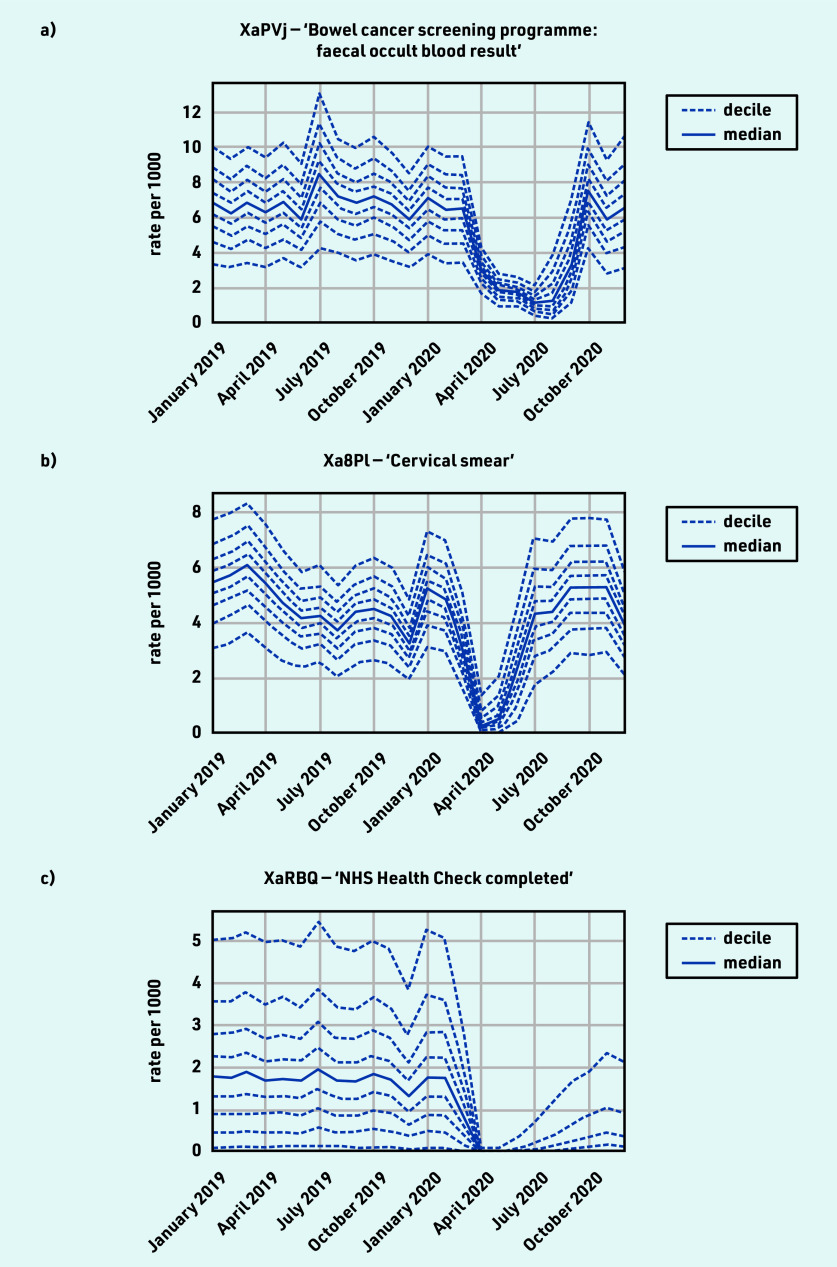
*Recording of CTV3 codes related to screening and related procedures across TPP practices in England (January 2019 to December 2020). a) ‘Bowel cancer screening programme: faecal occult blood result’. b) ‘Cervical smear’. c) ‘NHS Health Check completed’. Each CTV3 code does not necessarily represent all activity related to the description. Note: diabetic retinopathy screening was also included with screening codes but is discussed in the diabetes section.*

The majority of activity for screening and related procedures experienced a substantial decline, with recovery to slightly above normal levels by December 2020 (Table 1e), for example, ‘Bowel cancer screening programme: faecal occult blood result’ (April −52.1% and December +12.0%) and ‘Cervical smear’ (April ‒95.6% and December +20.9%). However, the patterns of recovery differed, with cervical smear screening levels near normal by July while bowel cancer screening was only beginning to recover around September (Figures 5a and 5b).

NHS health checks reduced from 1.8 per thousand in February to close to zero activity in April 2020 (median 0.0, with some recovery but median remaining zero by December 2020; Figure 5c and Table 1e).

### Processes related to medication

**Figure 6.**
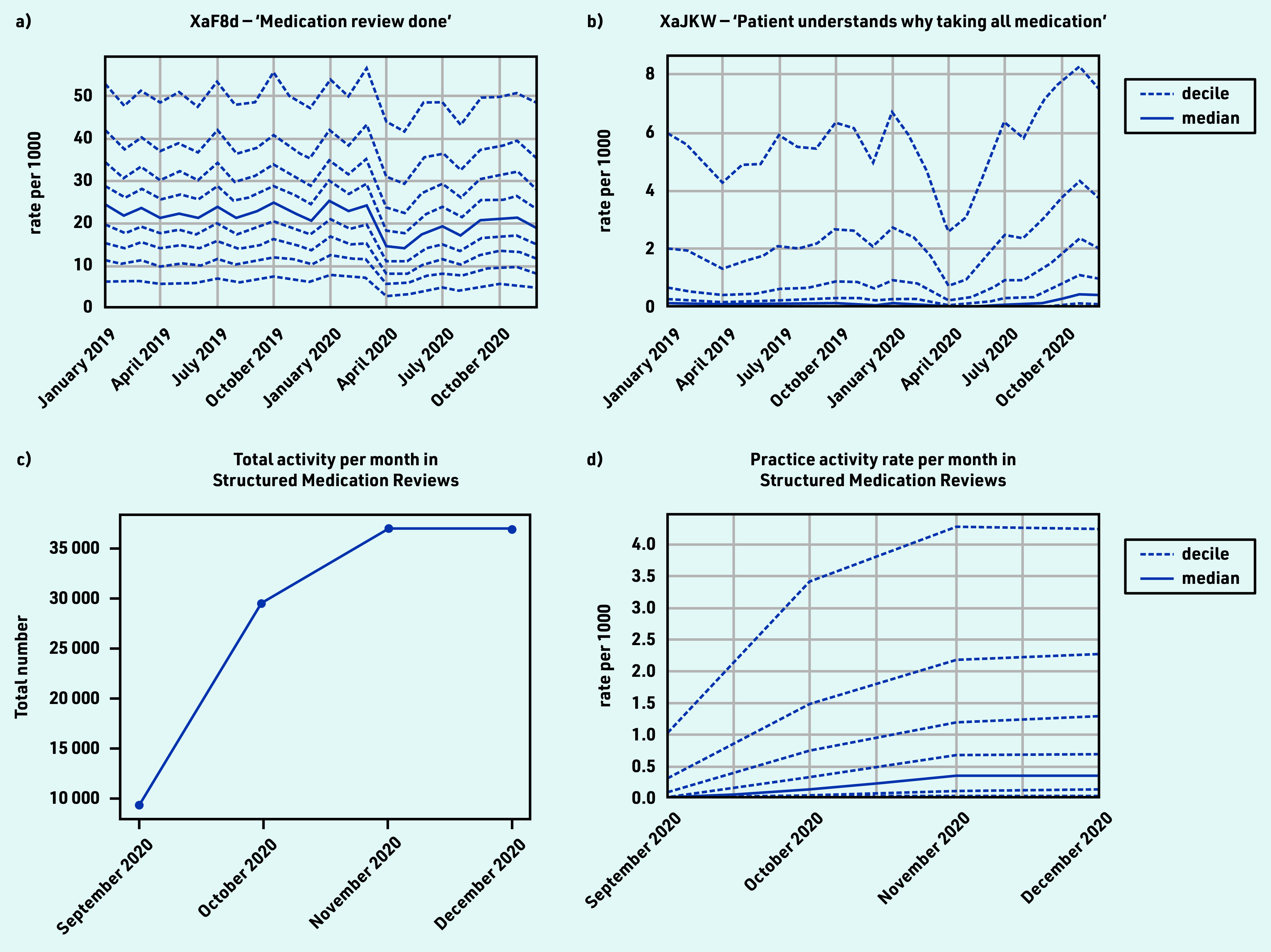
*Recording of CTV3 codes across TPP practices in England (January 2019 to December 2020). a) ‘Medication review done’. b) ‘Patient understands why taking all medication’. c) Total recording of Structured Medication Reviews (SNOMED-CT: 1239511000000100) across TPP practices in England throughout their period of use to date (September 2020 to December 2020). d) Practice deciles showing the rate per 1000 registered patients for Structured Medication Reviews. Each code is not necessarily an exhaustive collection of every activity related to the description.*

Several codes explicitly mentioned ‘medication review’, most commonly XaF8d ‘Medication review done’ (*n* = 7 250 000 occurrences; Supplementary Table S2f), which experienced a small dip and gradual recovery (April −31.0% and December −8.2%; Figure 6a and Table 1f). Other medication review codes (for example, review by pharmacist, for a specific disease, or indicating presence/absence of patient) were used less consistently between practices.

Several codes, generally uncommon but increasing in usage, were likely recorded as part of a medication review, such as ‘Patient understands why taking all medication’ (Figure 6b; February median 0.1, April 0.0, and December 0.4), and ‘Able to manage medication’ (Xa2yC) (see Supplementary Table S2f).

From September 2020 SMRs appeared (Figure 6c) and increased rapidly to around 35 000 records per month; however, some practices were recording them at much higher rates than others (Figure 6d and Supplementary Table S4).

## DISCUSSION

### Summary

This study identified not only widespread but also heterogeneous changes in clinical activity in primary care since the onset of the COVID-19 pandemic. There was generally good recovery by December 2020, with some exceptions: notably mental health, which showed minimal recovery. There was also variation from the median across practices, for both baseline and recovery. Further development of key measures of activity in primary care can support monitoring and evaluation of national policies around service restoration. The authors are now further developing the OpenSAFELY NHS Service Restoration Observatory for real-time monitoring of the key measures identified in this report.

### Strengths and limitations

The key strengths are the scale and completeness of the underlying raw EHR data, which were available close to real time, and the context provided by clinicians involved in the study. All processed data and analytical codes are openly available in the Supplementary Data or Github (https://github.com/opensafely/restoration-observatory-data-driven). The authors’ recommended key measures will be published in a live updating report, available at https://reports.opensafely.org, and they encourage other groups to use OpenSAFELY for further exploration. This data-driven approach is intended to generate an overall picture of primary care clinical activity, and explore high-volume areas that might otherwise be missed, for example, when not included in manually curated code lists.

Despite the strengths, there are some limitations as previously discussed in the authors’ earlier report.^18^ The data-driven approach and filtering processes may have omitted some relevant codes; codes do not necessarily indicate unique or new events and may be affected by changes in coding behaviour. All coded activity for patients registered at the end of the study period were included, and all activity was included under their latest practice. Patients who died or deregistered from TPP practices during the study period were not included. Overall, activity counts were up to 6%–8% lower than database totals in the earliest months of the study period.

### Comparison with existing literature

Given the diversity of clinical areas covered by this overarching analysis, the clinical advisory group evaluated and interpreted the variation for each clinical area separately.

#### Cardiovascular disease

Much of coded activity for CVD related to monitoring and remained around 40% reduced from pre-pandemic levels. This was not surprising because of changes in guidance and financial incentives.^21^ Electrocardiogram data presented in this study should be interpreted cautiously as these are often conducted outside primary care and not always systematically coded. QRISK2 is a commonly used risk assessment tool to identify people at increased risk of CVD, which helps to ensure appropriate treatment and reduce the risk of complications. The lack of recovery in activity of QRISK2 scores may have public health significance, potentially causing later diagnosis of heart disease and poorer early management, so continued monitoring of activity is important.^17^

Blood pressure monitoring (child code for ‘Examination of cardiovascular system’) is a high-volume activity that showed only partial recovery. Home blood pressure coding unsurprisingly increased; however, home monitoring in general may not always be recorded completely or consistently in general practice records. This is of particular interest because delays in the management of high blood pressure are associated with worse clinical outcomes.^22^ The consistent pattern of decrease in most cardiovascular-related activity is in line with results from other studies in the UK.^8^^,^^23^^,^^24^ The clinical advisory group proposed ‘blood pressure monitoring’ and ‘QRISK2 risk scores’ (or any cardiovascular risk score codes, including the newer QRISK3 codes) as key measures.

#### Diabetes

HbA1c is a long-term indicator of diabetes control; coding activity showed routine diabetes care almost entirely stopped, but largely rapidly recovered by December 2020. This is important as poor diabetic control can increase the risk of complications for patients living with diabetes.^25^ Diabetes monitoring and foot checks remained slightly below normal. In some areas, concerning foot changes may be seen by specialist services, hence may sometimes appear reduced in primary care. Diabetic retinopathy screening recovered less well; however, specialist clinics conduct this service and send reports to primary care that are manually coded; therefore, the sustained drop may indicate a coding change or other provider changes. Most diabetes care activity varied widely between practices, possibly due to differences in demographics and prevalence; coding (for example, use of data entry templates in EHR systems); use of external providers; or quality of care. The results from this study add to the findings from earlier studies that reported a rapid decline in the rate of new diabetes mellitus diagnoses and HbA1c testing in April 2020,^8^^,^^24^ and are in line with data showing good but incomplete recovery in the following months.^26^^,^^27^ It is therefore important to continue monitoring of routine diabetes care to ensure patients do not go undiagnosed or receive a delayed diagnosis. The clinical advisory group proposed HbA1c testing as a key sentinel measure.

#### Mental health

Most mental health activity coded by GPs showed a sustained reduction and this was consistent across various markers of activity. Previous research similarly showed that primary care-recorded diagnosis of common mental health conditions, and associated prescribing, reduced significantly in early 2020 and did not recover to pre-pandemic levels by the end of 2020.^8^^,^^24^^,^^28^ One region found a reduction in self-harm in primary care sustained through to May 2021.^29^ Other studies suggest that the impact on mental health may have been temporary but following a generally worsening trend,^30^ and the Department of Health and Social Care in England has responded by developing a targeted action plan.^31^ This was surprising given the much-discussed impact of the pandemic on mental health,^32^^,^^33^ but may be explained by patients either not seeking help or choosing other services or online resources, although the latter are unlikely to explain all the reduction. A recent study on mental health and telepsychiatry showed that the rapid shift to remote service delivery has not reached some groups of patients (in particular patients with dementia and mild cognitive impairment) who may require more tailored management.^34^ Dementia was not widely represented in the results of this study, perhaps being covered by a range of CTV3 codes; the authors plan to conduct further research on the impacts of COVID on dementia in primary care to capture this fully.

The reduction in ‘Depression interim review’ may warrant further investigation, but could reflect a change in coding behaviour; however, the similar reduction in codes for ‘Depressed mood’ would argue against this as the sole explanation. Nationally, the prescribing of antidepressants in primary care was sustained and continued the gradual increase observed before the COVID-19 pandemic (see Supplementary Figure S1).^35^ Further analyses are planned before proposing any single measure for immediate ongoing monitoring, as mental health activity (especially for depression and other mood disorders) spans different services such as community mental health trusts,^36^ which have limited coverage in OpenSAFELY.

#### Female and reproductive health

Clinical activity relating to female and reproductive health generally declined modestly around April 2020, with widespread recovery by December 2020. The reduction in contraception-related activity (discussion and monitoring) was likely explained by a combination of reduced need (use of non-prescription alternatives and less social contact), longer repeat prescriptions, check-ups being postponed, and long-acting reversible contraception (including coils and implants) not being fitted. Monthly contraceptive prescribing in England experienced only a small temporary reduction during the pandemic (see Supplementary Figure S2).^9^ Routine 6-week checks of infants were well maintained, likely prioritised as vital activities, possibly aided by increased use of telephone appointments and/or being carried out alongside 8-week immunisations, which were also prioritised.^37^ The slight increase in breast-related symptoms by December 2020 may indicate concerning delays in presentation. Current pregnancy records being slightly reduced may be explained by delayed presentation or increased use of self-referral directly to midwifery services. Other codes commonly recorded with pregnancy, for example, date of last menstrual period, would be reduced for the same reasons. There are also wider challenges in ascertaining the timing of pregnancies because of incomplete coding in EHRs.^38^^,^^39^ The low level of gestational diabetes indicates some codes for this condition were likely not captured here. Researchers have previously raised concerns about disruptions to sexual and reproductive healthcare services in the early stages of the pandemic.^40^ Although this study observed a decline in female and reproductive health activities in primary care, most activities returned to near or above pre-pandemic levels by December 2020. As there were relatively few codes capturing female and reproductive health and rapid recovery was observed in areas such as contraception, no key measures were suggested by the clinical advisory group.

#### Screening and related procedures

Maintaining screening activity is important to identify disease earlier.^41^ Some studies outside the UK have investigated the impact caused by disruption to screening services, and found evidence, for example, that new breast cancer diagnoses were reduced^42^ and some groups may have been affected more than others.^43^ This study shows that clinical activity related to screening declined substantially around April 2020 but there was widespread recovery by December 2020 to slightly above normal levels. For example, invitations to the NHS bowel screening service were paused in March 2020 and were subsequently issued at rates above 100% of normal levels.^11^ One exception to the recovery was NHS health checks, which were considered as ‘low priority’ in the Royal College of General Practitioners workload prioritisation.^37^ As a result of the widespread recovery, the clinical advisory group did not propose any measures for ongoing monitoring.

#### Processes related to medication

Medication reviews are structured, critical examinations of a patient’s medicine to optimise impact and minimise problems relating to medications.^44^ In September 2020, guidance on new SMRs was released, directing Primary Care Networks in England to identify patients who would benefit from an SMR.^19^^,^^45^^,^^46^ This study identified that processes related to medication, in particular medication reviews, were relatively well maintained during the pandemic, likely because of automated alerts commonly prompting clinicians when these are due. Uptake of SMRs was relatively rapid. Other related codes, such as ‘Patient understands why taking all medication’, are likely recorded during SMRs, which explains why they also increased. Not all practices were recording SMRs by January 2021, likely because pharmacists with the necessary training were not available in all practices. Use of this process was incentivised for 2021/2022 for certain patient groups,^19^ so, to monitor continuing changes in the coding of medication reviews, the clinical advisory group proposed a key measure comprising any medication reviews.

### Implications for research and practice

The COVID-19 pandemic brought new challenges for the NHS to deliver safe and effective routine care. To assess the consequences of the COVID-19 pandemic, it is important to consider its impact on the incidence, management, and outcomes of routine care. The post-pandemic period provides an opportunity to push health systems to be more resilient, responsive, and sustainable,^47^ offering an unprecedented natural experiment in new diagnoses and ongoing monitoring of patients’ conditions. Outcomes can be assessed to identify any clinical impacts on patients or tests that can be safely delayed without unintended impacts to free up healthcare capacity. The authors’ proposed NHS Service Restoration Observatory can support evaluation of national policies around service restoration and additionally provide opportunities for near real-time audit and feedback to rapidly identify and resolve concerns around health service activity. In particular, it is hoped that the data tools, such as the one described in this study, can be used to ensure continuity of high-priority clinical services during subsequent waves of the pandemic.

#### Future research

Across the clinical specialist areas, common themes for further research were identified (further detail on individual areas is provided in Supplementary Information S2). OpenSAFELY is a national data resource, and the authors encourage interested parties to consider exploring the following patterns in the platform:

1. Monitoring activity in granular groups such as those with established long-term conditions, those receiving certain medicines, or those receiving new diagnoses without prior history, to establish the impact on each group and on new diagnoses versus ongoing monitoring.
2. Extend monitoring of the chosen key measures of service restoration to encompass the entirety of primary care records available using OpenSAFELY, allowing federated analysis of >95% of patient EHR records in England.^48^
3. The level of ‘backlog’ could be analysed to inform NHS recovery plans and to establish whether those people who missed activities have later ‘caught up’ and whether there are some groups waiting longer than others. For example, some studies outside the UK have investigated the impact caused by disruption to screening services, and found evidence, for example, that new breast cancer diagnoses were reduced^42^ and some groups may have been affected more than others.^43^
4. The impact on cancer referrals could be analysed, including assessing any changes in cancer stage at diagnosis in those with relevant symptoms, or those who missed screening as a result of the COVID-19 pandemic.
5. Tackling health inequalities is a key part of the NHS Long Term Plan.^49^ The COVID-19 pandemic has highlighted disparities in how health care is delivered.^50^^,^^51^ Each clinical topic analysed in this report should be assessed in the context of health inequalities to explore whether impacts of the pandemic affected some groups more than others, and should take into account other activity, for example, prescriptions, referrals, and non-primary care activity.

## Data

Access to the underlying identifiable and potentially re-identifiable pseudonymised electronic health record data is tightly governed by various legislative and regulatory frameworks, and restricted by best practice. The data in OpenSAFELY is drawn from general practice data across England where TPP is the data processor. Bennett Institute developers and PIs (Helen J Curtis, Caroline E Morton, Sebastian CJ Bacon, Alex J Walker, William J Hulme, David Evans, Peter Inglesby, Simon Davy, George Hickman, and Ben Goldacre) holding contracts with NHS England have access to the OpenSAFELY pseudonymised data tables as needed to develop the OpenSAFELY tools.
